# Five good quality blastocysts retrieved via the physiological intracytoplasmic sperm injection dish HA binding method in a 50-year-old female

**DOI:** 10.11604/pamj.2022.42.196.35901

**Published:** 2022-07-12

**Authors:** Aasisjot Kaur, Akash More

**Affiliations:** 1 Department of Anatomy, School of Allied Health Science, Datta Meghe Institute of Medical Sciences, Sawangi, Wardha, Maharashtra, India,; 2 Department of Wardha Test Tube Baby Centre, Datta Meghe Institute of Medical Sciences, Sawangi, Wardha, Maharashtra, India

**Keywords:** Physiological intracytoplasmic sperm injection, in vitro fertilization, hyaluronan-specific receptor

## Image in medicine

Physiological Intracytoplasmic Sperm Injection (PICSI) is a scientific approach used to aid the embryologist in the selection of sperm in Intracytoplasmic Sperm Injection (ICSI), a specific kind of In Vitro Fertilization (IVF). A polystyrene culture dish with three microdots of hyaluronan adhered to the inside bottom serves as the PISCI sperm selection device. The cumulus coating of the oocyte (egg) contains hyaluronan, and the head of a mature sperm contains a hyaluronan-specific receptor that permits mature sperm to bind to hyaluronan. Immature sperm, on the other hand, do not bind. The PICSI Sperm Selection Device allows you to choose mature sperm based on their capacity to bind to a specific target hydrogel of hyaluronan. The PICSI Sperm Collection the Selection Device imitates the natural binding process transferring mature sperm to the oophorous cumulus, a crucial phase in the natural selection process fertilisation. After that, the sperm is deposited on a specific plate. This plate includes a few drops of a synthetic substance that coats the oocytes spontaneously, comparable to hyaluronic acid. The embryologist will be able to quickly identify those spermatozoa of acceptable quality and sufficient maturity that remain attached to these drops for microinjection of the oocytes.

**Figure 1:**
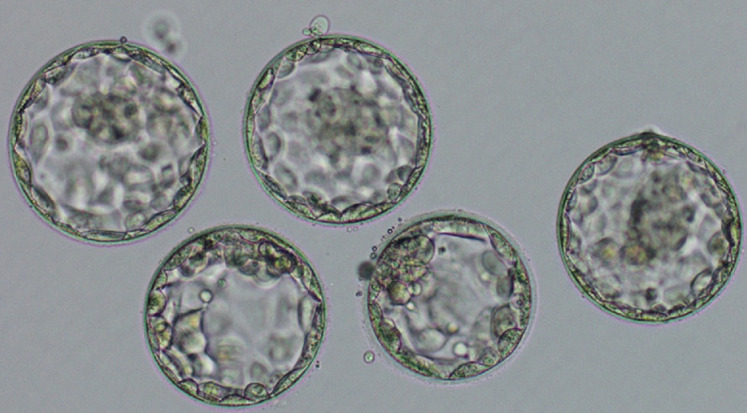
microscopic image showing 4AA blastocysts retrieved via the PICSI dish HA binding method

